# How Does Public Health Investment Affect Subjective Well-Being? Empirical Evidence from China

**DOI:** 10.3390/ijerph19095035

**Published:** 2022-04-21

**Authors:** Yingzhu Yang, Lexiang Zhao, Feng Cui

**Affiliations:** 1 Department of Economics, School of Economics, Peking University, Beijing 100871, China; yangyingzhu@pku.edu.cn; 2 China Investment Corporation Postdoctoral Center, Beijing 100010, China; 3 Department of Finance, PBC School of Finance, Tsinghua University, Beijing 100084, China; 4 Department of Public Finance and Taxation, School of International Trade and Economics, University of International Business and Economics, Beijing 100029, China; 201900130006@uibe.edu.cn

**Keywords:** public health investment, regional disparity, subjective well-being, individual relative deprivation index

## Abstract

Maximizing or improving residents’ subjective well-being is one of the basic purposes of public expenditure. As an important component of public expenditure, the impact of public health investment on residents’ subjective well-being receives considerable attention. Regarding the empirical evidence, this paper measures residents’ subjective well-being from the perspectives of overall cognitive happiness, life satisfaction, positive emotions and negative emotions, on the basis of a multi-level structural model of subjective well-being. Factor analysis is used to estimate the subjective well-being of residents at the province level in China, based on the China Family Panel Studies of 2018. In addition, structural equation modeling is employed to explore the impact of public health investment and its regional disparity on the subjective well-being of residents. The empirical results show that public health investment has a significant positive effect on residents’ subjective well-being. Moreover, there is an inverted U-shaped relationship between the regional disparity of public health investment and residents’ subjective well-being. Further study illustrates that the effects of public health investment and its regional disparity on residents’ subjective well-being are heterogeneous by group. Public health investment has a greater impact on the well-being of low- and middle-income, eastern and urban residents than high-income, midwest and rural residents.

## 1. Introduction

### 1.1. Background

After the Second World War, countries around the world began to adopt promoting economic growth as the main goal of their policies and systems. However, gross domestic product (GDP) could not reflect the social welfare state comprehensively. The blind pursuit of GDP could lead to environmental destruction, energy depletion and increased inequality, which has increasingly attracted the attentions of both academic and practice areas all over the world. How to maintain sustainable development is a matter of significant importance facing all countries. In this context, it is necessary to revalue the concept of sustainable development [1]. Many studies have proved that sustainability and subjective well-being are consistent [2,3]. The development of a view based on residents’ well-being is consistent with the connotation of sustainable development [4]. This reflects the further deepening and expansion of the previous understanding of development. Governments around the world increasingly attach importance to the improvement of residents’ subjective well-being. For instance, in the 1970s, Bhutan regarded gross national happiness as the measurement standard of social development instead of gross national product [5].

Subjective well-being is a kind of psychological experience which is both a factual judgment of objective conditions and a value judgment of the subjective meaning and satisfaction of life. It is the ultimate pursuit of human survival and development. Some studies show that subjective well-being is even more important than wealth [6]. With the development of society and living standards, residents’ pursuit of well-being is becoming more and more urgent. In fact, the pursuit of subjective well-being is not entirely a personal matter, and the government has an irreplaceable role to play. Focusing on improving people’s subjective well-being is an important embodiment of concern for people’s livelihood and human value. Maximizing or raising residents’ subjective well-being is considered as one of the basic objectives of the government [7].

Public health investment is one of the important policy means for the government to provide public services and improve residents’ subjective well-being. With increasing population aging and the improvement of economic development, public health investment continues to expand and the demand for health continues to increase. As an important component of public expenditure, the impact of public health investment on residents’ subjective well-being receives considerable attention. On one hand, public health investment could reduce private medical consumption which indirectly improve residents’ income. On the other hand, public health investment would increase residents’ sense of security and reduce the anxiety related with health problems. Public expenditure consistent with residents’ demand for public goods will best promote residents’ subjective well-being. Therefore, how public health investment and its disparity influence the subjective well-being of residents is an important theoretical and practical issue.

What is the relationship between public health investment and residents’ subjective well-being? How does the regional disparity in public health expenditure affect residents’ subjective well-being? Is there any difference in the impact of public health investment on the subjective well-being of different groups? Research on these issues will provide a theoretical basis and empirical evidence for the optimization of public health investment and promote the positive role of public health investment in improving residents’ subjective well-being.

This paper uses the micro data of the China Family Panel Studies 2018 (CFPS2018) to match the provincial public health expenditure data to investigate the impact of public health investment and its disparity on residents’ subjective well-being. The measurement of subjective well-being is based on the Multi-Level Structure Model of Subjective Well-Being [8]. This paper measures residents’ subjective well-being from four perspectives: overall cognitive happiness valuation, life satisfaction, positive emotions and negative emotions. A factor model is employed to estimate the level of residents’ subjective well-being. In addition, the Multiple Indicators and Multiple Causes Model (MIMIC model) is used to study the impact on residents’ subjective well-being of public health investment and its regional disparity.

This paper includes five sections and is organized as follows. Section 1 presents the research background, literature review and theoretical analysis. Section 2 introduces the data and methodology. Section 3 presents the empirical results of both the factor model and MIMIC model. Section 4 discusses the empirical research conclusions, policy implications and limitations of this paper. Section 5 gives the summary and conclusions of the study.

### 1.2. Literature Review

The research on subjective well-being began in the 1950s with quality-of-life research and the positive psychology movement. After the Easterlin Paradox was proposed in the 1970s [9], the theoretical and empirical research on subjective well-being has gained more attention from economic scholars. The Easterlin Paradox states that there will be no obvious positive correlation between income and happiness when national income reaches a certain level. Initially, happiness economics mainly explored the micro factors that affect the subjective well-being of the public. Demographic characteristics including personal income, gender, age, education level, marital status and health status all affect residents’ subjective well-being [10,11,12]. Later, macroeconomic variables were gradually introduced into the research [13]. Social characteristics such as economic factors, political factors and cultural factors also have an impact on subjective well-being [14,15,16,17].

According to the relevant research, influencing factors of subjective well-being can be divided into micro level and macro level [18]. Micro influence factors refer to the sociodemographic factors such as age, sex, marriage, health and psychology. Some studies have found that people’s subjective well-being tends to improve as they get older [19]. Other scholars have found that there is a U-shaped relationship between age and subjective well-being [20]. Different scholars hold different views on the impact of gender on subjective well-being. Most studies believe that women’s subjective well-being is higher than men’s [21]. However, some researchers have pointed out that as women’s social role has transformed, women need to juggle family duties and their careers. This means that women are under more stress than men, which leads to lower subjective well-being [22]. Usually, people with stable marriages are happier than others [23]. A harmonious family relationship significantly improves people’s subjective well-being [24]. People with a higher education level are more competitive for better employment opportunities and higher income, which leads to higher subjective well-being [25]. In addition, both the absolute and relative income level affect residents’ subjective well-being [26].

Macro influence factors include the system, policy, government expenditure and other social economic factors. In addition to microeconomic variables, macroeconomic variables, such as unemployment and inflation, also affect subjective well-being. Both unemployment and inflation have a great negative impact on residents’ subjective well-being, which has been confirmed by scholars’ studies [21,27]. Moreover, social capital may also improve subjective well-being [18]. Later, some scholars discussed the influence of environmental pollution, social security, urbanization and other factors on residents’ subjective well-being [28,29].

In recent years, as an important explanation of the Easterlin Paradox, public expenditure has attracted more and more attention. Research on the impact of public expenditure on residents’ subjective well-being has basically reached a consensus. Most researchers believe that government expenditure is conducive to improving residents’ subjective well-being. The government plays a key role in social and economic development, and residents’ subjective well-being is the focus and ultimate goal of the government. To a certain extent, the loss of subjective well-being caused by the bandwagon effect (the effect of people comparing themselves with others) could be reduced by transforming highly competitive private consumption into public goods consumption available to all [30,31]. Meanwhile, with the improvement of the economic level, residents’ demand for medical and health services has gradually increased. Public health investment is conducive to releasing residents’ consumption demand and improving residents’ subjective well-being [32].

A large number of studies on public health investment and residents’ subjective well-being believe that there is a positive correlation between the two. Bjrnskov et al. (2003) used cross-sectional data of 74 countries to explore the relationship between public health investment and residents’ subjective well-being, and the results show that public health investment had a significant positive impact on residents’ subjective well-being [33]. Dutt et al. (2006) believed that government expenditure on education, health, environmental protection and safety can improve residents’ happiness [34]. Helliwell and Huang (2008) pointed out that public health investment has a much greater effect on the well-being of low-income groups than high-income groups [35]. Kotakorpi and Laamanenz (2010) found that a higher public health investment is conducive to improving residents’ subjective well-being in Finland, and middle-income groups prefer a higher public health investment compared with low-income and high-income groups [36]. Lu Yuanping and Zhang Kezhong (2010) used World Values Data to study the impact of pro-poor public expenditures on residents’ subjective well-being in China. The results show that public expenditures on health, education and social security all play a significant role in promoting residents’ subjective well-being, and public health expenditure plays the most significant role [37]. Hu Hongshu and Lu Yuanping (2012) used CGSS data to study the influence of public expenditure on farmers’ subjective well-being. The research results show that pro-poor public spending significantly improves farmers’ subjective well-being and public health investment has the greatest impact on farmers’ subjective well-being. Moreover, the public health investment has the greatest effect on the subjective well-being of low-income farmers [32].

Other studies propose a non-linear or negative relationship exists between public health investment and residents’ subjective well-being. Hessami (2010) found that there is a U-shaped relationship between public health expenditure and residents’ well-being by analyzing the relationship between the composition of public expenditure and residents’ well-being [38]. Lok-Sang and Yew-Kwang (2016) found that there is an inverted U-shaped relationship between public health expenditure and residents’ life satisfaction, which means there is an optimal public health expenditure [39]. According to Tang Fenglin and Lei Pengfei’s (2014) research, public health expenditure slightly reduces residents’ well-being due to low capital utility [40].

Overall, most studies suggest that government health expenditure is conducive to the improvement of residents’ subjective well-being, while a few propose that government health expenditure reduces residents’ subjective well-being due to the low efficiency of health resource allocation and the conflicts between doctors and patients. More and more scholars have paid attention to the impact of public health investment on residents’ subjective well-being. However, most of the existing research just regards public health investment as a component of government expenditure and briefly discusses the relationship between public health investment and residents’ subjective well-being. Few studies explore the relationship between the two independently. Meanwhile, existing studies often choose an explicit proxy variable for subjective well-being. However, subjective well-being is a subjective concept, which should be measured from multiple dimensions. Only using one explicit variable to measure subjective well-being cannot reflect the whole picture of subjective well-being and would inevitably lead to corresponding measurement errors. In order to understand subjective well-being more comprehensively, this paper uses a factor model to construct a latent variable on the basis of the multi-level structural model of SWB. In addition, the MIMIC model is used to examine the impact of public health investment and its disparity on subjective well-being. This compensates for the lack of existing literature and provides a new perspective for future research.

### 1.3. Theoretical Analysis

Well-being has always been a major concern in psychology, philosophy, sociology and many other research fields. It carries rich connotations. It is meaningful to discuss the notion and connotations of well-being from the perspective of economics. In 1974, the Easterlin Paradox was proposed. After this, subjective well-being has finally become an important research issue in economics. However, the relation between subjective well-being and economics can be dated to Bentham’s utility theory. Bentham thought people’s action is motivated solely by pleasure and pain. Pleasure is actually a kind of embodiment of well-being, which means that well-being is a property of utility. With the rise of ordinal utility theory, economics focuses more on the individual’s choices, so the connotation of well-being contained in utility is gradually lost. Neoclassical economists believe that utility is the satisfaction obtained from the consumption of certain goods and services, which equates utility simply with the pursuit of material benefits. According to Neoclassical economics theory, individuals are rational enough to make choices which could maximize their utility. To some extent, this is reasonable but deviates from a great deal of economic phenomena. Individuals are actually far from rational economic people who simply pursue the maximization of material benefits. In 1997, Kahneman proposed prospect theory which divides utility into decision utility and experienced utility. The former refers to the degree of importance of one choice to other choices, which corresponds to the desirability implication of utility emphasized by neoclassicists. The latter refers to the pleasure experience brought by a certain choice, which is consistent with the concept of utility proposed by classical economists. In the framework of economics, well-being belongs to experienced utility so it can be measured. Now, more and more scholars use subjective well-being data as the proxy for experienced utility [41]. As the embodiment of an individual’s satisfaction, well-being is influenced by the difference between the realistic and the expected results. This means that individuals’ well-being (satisfaction) does not only depend on the absolute level but also on the relative level of the results of an event (the difference or change in the result relative to a reference point).

Well-being is a subjective psychological experience. Many psychologists study well-being from the subjective spiritual level of individuals, which is called subjective well-being (SWB). Subjective well-being refers to an individual’s holistic evaluation of his or her life quality based on self-defined standards [42], which reflects an individual’s emotional response and life satisfaction over a long period of time. Subjective well-being is mainly composed of a cognitive component and an emotional component. The cognitive component refers to life satisfaction, which is the attitude and feeling after comparing the actual life state with the ideal life state [6]. The emotional component consists of positive emotions and negative emotions. Subjective well-being embraces three characteristics: subjectivity, stability and integrity. Subjectivity refers to the evaluation of subjective well-being being completely dependent on the individual’s own standards. That is to say, whether individuals are happy or not depends entirely on how they evaluate their life subjectively and their subjective feelings. Integrity means that the measurement of subjective well-being is a kind of comprehensive evaluation. Stability means that subjective well-being measures long-term emotional response and life satisfaction and is a relatively stable value.

The multi-level structure model of SWB [8] divides SWB into three levels and four areas specifically: the first level is the overall SWB, which is the overall evaluation of life quality; the second level includes general life satisfaction (overall judgment of individual life) and satisfaction in important areas of life (such as job satisfaction); the third level is the embodiment of the second level, such as positive emotions including pleasure and happiness, and negative emotions including sadness, and life satisfaction in important areas including job satisfaction. Generally speaking, the higher the individual’s overall satisfaction with their quality of life, and the more positive emotions and fewer negative emotions are experienced, the higher the individual’s subjective well-being will be.

## 2. Materials and Methods

### 2.1. Data and Methodology

The data used in this paper are from the China Family Panel Studies (CFPS) of 2018. The CFPS are a national, large-scale, multidisciplinary social tracking survey project launched by the Institute of Social Science Survey of Peking University. This project aims to collect data at individual, family and community levels through a questionnaire survey to reflect the changes in China’s economy, society, population, education and health. The study has been approved by the Peking University Biomedical Ethics Review Committee (Approval No. IRB00001052-14010). The sample covers 25 provinces in China, which accounts for more than 95% of the country’s population. Therefore, the data are nationally representative [43]. There is a variable which reveals the code of the province to which the interviewee belongs. We matched the provincial public health expenditure data with CFPS micro data “1:m” (one to more) by the province code.

According to the theoretical analysis, this paper measures residents’ subjective well-being from four perspectives: overall cognitive happiness valuation, life satisfaction, positive emotions and negative emotions. This paper uses the answers to the following six questions as the measurement indexes, respectively (see Table 1). Negative indicators are turned into positive indicators before factor analysis. The measurement equation of subjective well-being is as follows:

(1) $$\left\{\begin{array}{c} x_{1}=\alpha_{1}+X\beta_{1}+e.x_{1}\\ x_{2}=\alpha_{2}+X\beta_{2}+e.x_{2}\\ x_{3}=\alpha_{3}+X\beta_{3}+e.x_{3}\\ x_{4}=\alpha_{4}+X\beta_{4}+e.x_{4}\\ x_{5}=\alpha_{5}+X\beta_{5}+e.x_{5}\\ x_{6}=\alpha_{6}+X\beta_{6}+e.x_{6} \end{array}\right.$$

where $x_{1}$–$x_{6}$ denotes six measurment indexes of subjective well-being, respectively, X denotes the latent variable subjective well-being, and $\Lambda =\left\{\beta_{1},\beta_{2},\beta_{3},\beta_{4},\beta_{5},\beta_{6}\right\}$ denotes the factor load matrix.

Then, this paper uses the Multiple Indicators and Multiple Causes Model (MIMIC model) to examine the relationship between residents’ subjective well-being and public health investment. In essence, the MIMIC model is a special structural equation model (SEM model), whose independent variables are observed variables, and dependent variables are latent variables. With both cause indicators and outcome indicators, the MIMIC model can examine the impact of all cause indicators and outcome indicators on the latent variables. It is mainly composed of a structural equation and a measurement equation. The core explanatory variables are public health investment and its disparity. Public health investment is measured by per capita public health investment. The disparity of public health investment is measured by the individual relative deprivation index. Assuming Y is a group; the sample size is n∈N={1, 2, 3, ⋯, n}; $y=\left(y_{1},\;y_{2},\;y_{3},\;\cdots ,\;y_{n}\right)$ is the indicator vector of per capita public health investment; with $y_{1}\leq y_{2}\leq y_{3}\leq \cdots \leq y_{n}$; $y_{i}$ and $y_{j}$ being the per capita public health investment of province i and j, respectively, where i, j∈N.

The computational formula of the Yitzhaki individual relative deprivation index is as follows:

(2) $$D\left(y_{i},\;y_{j}\right)=\{\begin{array}{c} y_{i}-y_{j},if\;y_{i}>y_{j}\\ \;\;\;\;\;\;0,\;if\;y_{i}\leq y_{j} \end{array}$$

According to Equation (2), we can obtain the individual relative deprivation index of i in group Y:

(3) $$D\left(y_{i},\;y\right)=\frac{1}{n}\underset{y_{i}>y_{j}}{\sum }\left(y_{i}-y_{j}\right)$$

Then, the deviation standardization method has been employed to make the index value dimensionless as in Equation (4).

(4) $$D\left(y_{i},y\right)=\frac{1}{n\left[\max\left(y\right)-\min\left(y\right)\right]}\;\underset{y_{i}>y_{j}}{\sum }\left(y_{i}-y_{j}\right)$$

where max(y) is the maximum value of *Y* and min(y) is the minimum value of *Y*. The higher the $D\left(y_{i},\;y\right)$ is, the greater the regional difference of per capita public health investment.

In addition to the above-mentioned variables, residents’ SWB may also be affected by personal and family characteristics [44]. In order to fully consider the influence of other factors on residents’ SWB, this paper selects corresponding control variables according to relevant studies [15,45,46]. Among them, residents’ personal characteristic variables include age and its square, gender, household registration, nationality, marital status, education, political affiliation, health, relative personal income and social status. The family characteristic variables include per capita household income, family social network and family relationship. Specific variable definitions are shown in Table 1.

The summary statistics of sample data and sample distribution of key variables are shown in Table 2 and Table 3 respectively. On the basis of the original data, a total of 23,031 valid samples are obtained in this paper, after eliminating the samples with missing key variables and non-logical relationships. The specific composition is as follows: 11,385 men and 11,646 women; 16,917 in rural areas and 6114 in non-agricultural areas; 20,902 ethnic Han and 2129 ethnic minorities; 2985 married and 20,046 non-married (including unmarried, cohabiting, divorced and widowed); 2129 members of the Communist Party of China and 20,698 not; the average years of schooling is 7.63; the average age is 47.89 years old, with the youngest being 16 years old and the oldest 96 years old; the average subjective social status evaluation is 3.13; the average self-reported health level is 3.13; the mean of the relative income evaluation is 2.93; the average per capita annual household income is CNY 18,973.01; the average annual expenditure of human gift is CNY 5072.27; the average family relationships (weekly frequency of having dinner with family members) is 5.85.

### 2.2. Model

#### 2.2.1. Factor Model

**Figure 1:**
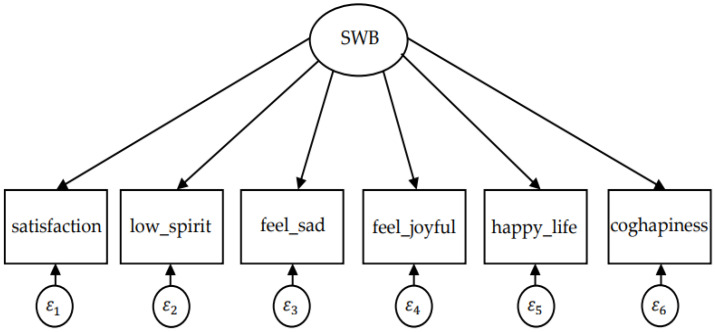
Factor model of “subjective well-being”.

As shown in Figure 1, this paper uses six observed variables to measure subjective well-being. Subjective well-being is a latent variable which represents the common factor of the six observed variables. $\varepsilon_{1}–\varepsilon_{6}$ denotes error terms representing the unique variance of each indicator. First, exploratory factor analysis (EFA) is used to test whether there is a unique common factor among the indicators to ensure that the six indicators could measure “subjective well-being” from different aspects without creating redundant factors. Then, confirmatory factor analysis (CFA) is used to test the fitting degree and feasibility of the entire factor model.

#### 2.2.2. MIMIC Model

In this paper, the endogenous latent variable is residents’ subjective well-being and the model is set as follows:

(5) $$\eta =\gamma_{1}x_{1}+\gamma_{2}x_{2}+\cdot \cdot \cdot +\gamma_{n}x_{n}+\zeta$$

(6) $$y_{1}=\lambda_{1}\eta +\varepsilon_{1},\;y_{2}=\lambda_{2}\eta +\varepsilon_{2},\cdot \cdot \cdot ,y_{m}=\lambda_{m}\eta +\varepsilon_{m}$$

**Figure 2:**
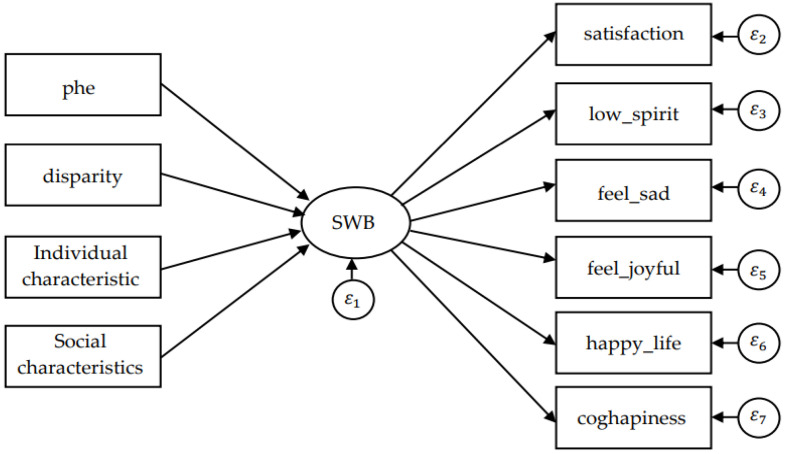
Path diagram of MIMIC model.

Equation (5) is the structural equation which describes the relationship between subjective well-being and cause variables. η is a latent variable which represents subjective well-being. $x^{\prime }=\left(x_{1},x_{2},\ldots ,x_{n}\right)$ is the exogenous observed variable vector, representing the main cause of subjective well-being; $\Lambda_{y}=\left(\lambda_{1},\lambda_{2},\ldots ,\lambda_{m}\right)$ is the factor load matrix; and ζ is the random error term of the structural equation. Equation (6) is the measurement equation which describes the specific path for measuring the latent variable (Acock, 2013 [47]; Schumacker and Lomax, 2004 [48]); $y^{\prime }=\left(y_{1},y_{2},\ldots ,y_{n}\right)$ is the endogenous observed variable vector; $\Gamma =\left(\gamma_{1},\gamma_{2},\ldots ,\gamma_{n}\right)$ is the coefficient matrix of the impact of exogenous variables on endogenous latent variables; $\varepsilon =\left(\varepsilon_{1},\varepsilon_{2},\ldots ,\varepsilon_{m}\right)$ is the error term of the endogenous variables. Based on the model set above, the path diagram of the MIMIC model is shown in Figure 2.

## 3. Results

### 3.1. Measuring Result of Factor Model

#### 3.1.1. Exploratory Factor Analysis

Before factor analysis, a series of prior tests, including the Bartlett test, the KMO test and the Cronbach reliability test, should be performed to determine whether the factor analysis method is applicable. The result of the Bartlett test is χ^2^ = 34,236.65 (*p* = 0), which rejects the null hypothesis that there is no correlation between the measurement indicators. The result of the KMO test is 0.70 and the coefficient of the Cronbach reliability test is 0.71, indicating that the CFPS questionnaire data are highly reliable. The three test results are all within the critical value, indicating that the specification of the factor model of subjective well-being is reasonable.

On the basis of the prior tests, exploratory factor analysis is further used to test whether there is only one common factor among the six selected indicator variables. Traditional principal component analysis (PCF) assumes that each indicator does not have its own unique variance, which is inconsistent with reality [47]. In this paper, the principal axis factor method (PF), the iterative principal axis factor method (IPF) and the maximum likelihood factor method (MLF) are used for exploratory factor analysis. The specific results are shown in Table 4. The analysis results show that there is only one factor which has an eigenvalue greater than 1, calculated by PF, IPF and MLF. Only when the eigenvalue is greater than 1 can the factor be retained. These analysis results show that there is only one common factor among the six indicators, which is subjective well-being.

#### 3.1.2. Confirmatory Factor Analysis

In order to test whether the factor load coefficient of the whole factor model and each measurement indicator of subjective well-being are significant, confirmatory factor analysis (CFA) is used for further analysis and test. This paper uses Maximum Likelihood with Missing Values (MLMV) for estimation. The estimation results show that the load coefficients of the six indicators of the factor model are significant. In addition, the comparative fitting index (CFI), the model fitting coefficient (R^2^(CD)) and the root mean square of approximate error (RMSEA) are used to test the fitting degree of the factor model. Table 5 reports the test results. The CFI is 0.999 and the RMSEA is 0.016. All of these indicators are up to the judgment criteria, which indicates that the factor model designed in this paper has an ideal fitting effect on the actual data.

### 3.2. Measured Value of Subjective Well-Being

Based on the estimated results of the confirmatory factor analysis above, the value adjustment factor of subjective well-being is 11.76, which is 77.53% of the maximum 15.17. Table 6 reports the measured values of subjective well-being of the residents in each province. The first column shows the value adjustment factors and the second column reports the standardized values of subjective well-being calculated by the efficacy coefficient method.

**Figure 3:**
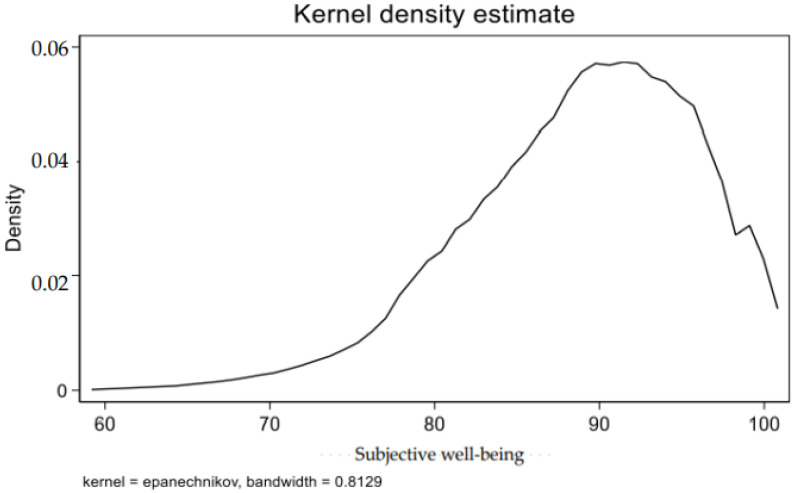
Kernel density curve of subjective well-being.

According to Table 6, the provinces with the highest subjective well-being are Shandong, Shanghai and Liaoning, in 2018. Among the twenty-five provinces covered by the CFPS survey, fifteen provinces’ subjective well-being of residents are higher than the national average and ten are lower than the national average. And the kernel density curve of subjective well-being is shown in Figure 3.

### 3.3. Estimation Results of Basic MIMIC Model

Both the ML method and the MLMV method are used, and the regression results are robust. In Table 7, the regression results shown in Columns (1), (2) and (3) are obtained by ML, ML with robust standard error and MLMV with robust standard error, respectively. The following analysis is based on the estimation results of the ML method. Part B is the estimation result of the measurement equation. Part C shows the fit index of the MIMIC model. The root mean square error (RMSEA) is 0.035. The CFI is 0.901 and the fit coefficient R^2^(CD) of the whole model is 0.365. These results indicate that the overall fitting results of the MIMIC model set in this paper are acceptable.

Part A shows the estimation result of the structural equation. In column (1)–(3), the regression coefficient of public health investment is significantly positive, which indicates that there is a significant positive relationship between public health investment and residents’ well-being. This is probably because the increase in public health investment would reduce residents’ private healthcare consumption pressure, which will affect residents’ subjective well-being through the following two channels. On one hand, this means providing indirect support to residents’ consumption and reducing precautionary savings, which would reduce their uncertain expectation and narrow the income gap. On the other hand, to some extent this can alleviate the negative effect of comparison consumption on residents’ subjective well-being. In column (1)–(3), the coefficient of the primary term of disparity is significantly positive and the coefficient of the quadratic term is significantly negative. That means that there is an inverted U-shaped relationship between the disparity of public health investment and residents’ subjective well-being, with an inflection point of 0.247.

The regression results of the control variables are basically consistent with previous studies. There is an inverted U-shaped relationship between age and subjective well-being which is consistent with the research results of Hawkes (2012) [49]. Compared with young and old people, middle-aged people bear more pressure from family and career, so their subjective well-being is relatively low. Men’s subjective well-being is significantly higher than women’s, which may be mainly due to the transformation of women’s social role. Modern women need to juggle family duties and their career which means that women are under more stress than men, which is consistent with the research conclusion of Graham and Felton (2006) [22]. Human capital can improve residents’ subjective well-being, which is mainly manifested in education and health. Residents with a higher education level and higher health level have higher subjective well-being. Marriage helps to improve residents’ subjective well-being, and both the absolute income level and relative income level have a significant positive impact on residents’ subjective well-being. The improvement of social status is conducive to the improvement of residents’ subjective well-being, which is consistent with the research conclusion of Burr et al. (2011) [50]. Harmonious family relationships can also improve residents’ subjective well-being. As shown in the regression results, residents who have dinner with their families more often have higher subjective well-being. Both family economic status and family social network are conducive to the improvement of residents’ subjective well-being, which is consistent with the findings of Anderson et al. (2001) [51].

### 3.4. Further Heterogeneity Analysis

According to the per capita household income of residents, the sample is divided into five income groups: low-income, low–middle-income, middle-income, upper middle-income and high-income. The household income per capita is ranked from low to high. The residents with per capita household income of less than CNY 5000 are classified as the low-income group; the residents with per capita household income of more than CNY 5000 and less than CNY 10,000 are divided into the low–middle-income group; those with a per capita household income of more than CNY 10,000 and less than CNY 16,666.67 are classified as the middle-income group; those with per capita household income of more than CNY 16,666.67 and less than CNY 30,000 belong to the upper middle-income group; and those with a per capita household income of more than CNY 30,000 are classified as the high-income group.

Table 8 shows the estimation results of subsample regressions by different income groups. The results indicate that public health investment has a significant role in promoting the subjective well-being of different income groups. It can be found that public health investment has a stronger effect on the subjective well-being of residents in low- and low–middle-income groups than those of high-income groups. This suggests that the increase in public health investment is more conducive to alleviating the medical problems of low-income groups. With limited incomes, low-income groups cannot afford excessive medical expenses which makes them more dependent on public health investment. Therefore, public health investment has a greater effect on improving their subjective well-being.

In column (1)–(4), the coefficient of the primary term of disparity is significantly positive and the coefficient of the quadratic term is significantly negative. Thus, there is an inverted U-shaped relationship between the disparity of public health investment and residents’ subjective well-being in different income groups, except the high-income group. The inflection points of the low-, low–middle-, middle-, upper middle- and high-income groups are 0.256, 0.232, 0.198, 0.267 and 0.411, respectively. The high-income group’s subjective well-being could not be affected by the regional disparity in public health investment. In contrast, the middle-income group has the lowest tolerance for the disparity. The demand and consumption of medical and health services of the high-income groups do not depend on the public health investment. They have greater freedom to choose medical services beyond space limitations. For them, the cost of seeing a doctor in regions with richer medical resources and higher medical service quality is completely affordable, so they have a higher tolerance for regional disparity. Those in the low-income groups pay more attention to the absolute level of public health investment than to regional disparity, so they have a relatively high tolerance for regional disparity.

According to the provinces where residents live, the sample is divided into two sub-samples of eastern and midwest regions. Regressions are carried out to investigate the impact of public health investment on residents’ subjective well-being in these different regions. The regression results are shown in Table 9. Public health investment has a significant promoting effect on the subjective well-being of the residents in both the eastern and midwest regions. Moreover, the promoting effect is higher in the eastern regions than in the midwest regions. The main reason is that the eastern regions are economically developed, and the total amount of public health investment is significantly greater than that of the midwest regions, which makes the effect of public health investment stronger. There is an inverted U-shaped relationship between the disparity of public health investment and residents’ subjective well-being both in the eastern and midwest regions, with inflection points of 0.233 and 0.187, respectively. It can be seen that the regions with stronger economic power are more tolerant of regional disparity in public health investment.

The sample is divided into rural and urban residents according to the registered permanent residence. Regressions are carried out to investigate the difference in the impact of public health investment and its disparity on the subjective well-being of rural and urban residents. The regression results are shown in Table 9. The regression results show that public health investment significantly promotes the subjective well-being of both rural and urban residents. Compared with urban residents, the regression coefficient of public health investment in rural areas is larger. In recent years, public health investment gradually tilts towards rural areas which brings a greater effect on improving the subjective well-being of rural residents. In both the rural and urban samples, there is an inverted U-shaped relationship between regional disparity in public health investment and residents’ subjective well-being, with inflection points of 0.228 and 0.307, respectively. It is obvious that urban residents have a stronger tolerance for regional disparity in public health investment.

## 4. Discussion

### 4.1. Results Analysis and Policy Implication

Studies on happiness economics provide clear policy implications and a basis for testing policy effects. With the increasing demand for health, public health investment plays an increasingly important role in meeting the medical needs and improving the overall health level of residents, which are closely related to residents’ subjective well-being. It is of great theoretical and practical significance to study the relationship between public health investment and residents’ well-being.

The first basic conclusion of this paper is that there is a significantly positive relationship between public health investment and residents’ subjective well-being, which is consistent with the conclusions of Ram (2009) and Kim and Kim (2012) [52,53]. According to goal theory, when an individual’s goals or needs are met, it induces a sense of well-being in people. Public health investment is conducive to meeting the growing health needs of residents which would improve residents’ subjective well-being. The second conclusion is that there is an inverted U-shaped relationship between the disparity of public health investment and residents’ subjective well-being. On the basis of social comparison theory, when an individual realizes that his own situation is worse than that of other members of the social group, his subjective well-being will decrease. Moreover, the greater the perceived gap between the individual and others, the lower his subjective well-being will be. This is consistent with the relative deprivation theory. When the regional disparity of public health investment exceeds a certain limitation of tolerance, the subjective well-being of residents will decrease significantly. Moreover, through heterogeneity analysis, it is found that there are regional differences in the impact of public health expenditure and its regional disparity on residents’ subjective well-being in different groups. Public health expenditure has a greater effect on the subjective well-being of residents in low- and middle-income groups than those in high-income groups. The reason is that the poor have lower incomes and lower consumption levels, making them more dependent on government spending. Public health expenditure has a greater impact on residents’ subjective well-being in east China than in midwest China. This is probably because the total public health investment in the eastern region is significantly larger than that in the central and western regions, which has a stronger promoting effect on residents’ subjective well-being. In addition, from the perspective of household registration, public health investment plays a stronger role in promoting the subjective well-being of rural residents. The reason may be that the marginal welfare effect brought by the same public health expenditure is greater than that for urban residents because the income level in rural areas is relatively low.

With the economic development of various countries, health investment has played a more and more important role in residents’ subjective well-being. The government should continue to expand health investment to promote residents’ well-being. In particular in order to promote social equity, the government should further increase health investment in rural areas and poor areas to improve the subjective well-being of different groups. Then, the medical insurance system should be improved. In addition, the government should pay attention to the needs of the low- and middle-income groups for medical and health services and ensure the basic medical needs of residents are met.

### 4.2. Limitations

The paper is not without limitations. Limited by the data available, the empirical analysis uses the provincial public health expenditure data to investigate the impact of public health and its disparity on residents’ subjective well-being. In the future, if the county or city data could be used, the research conclusions may be more comprehensive and accurate. Moreover, the paper is limited by the method; the research design of this paper is cross-sectional which infers correlations rather than casual relationships between variables. This issue should be further studied by improving the research method in the future. Meanwhile, the political system and medical system could have an impact on public health investment. It is worth noting that the extent to which government health expenditure replaces individual health expenditure is different among countries. Healthcare provided by public health mainly protects the vulnerable groups, which plays an irreplaceable role in promoting social equity.

## 5. Conclusions

This paper measures residents’ subjective well-being from four dimensions: overall cognitive happiness, life satisfaction, positive emotions and negative emotions, on the basis of the multi-level structural model of SWB. Factor analysis is used to estimate the subjective well-being of residents at the province level in China, based on the China Family Panel Studies 2018. In addition, the MIMIC model has been employed to explore the impact of public health investment and its regional disparity on residents’ subjective well-being. The empirical results show that there is a significant positive relationship between public health investment and residents’ subjective well-being. Moreover, there is an inverted U-shaped relationship between the regional disparity of public health investment and residents’ subjective well-being. Further heterogeneity analysis shows that the effects of public health investment and its regional disparity on residents’ subjective well-being is heterogeneous by group. Specifically, public health investment has a greater impact on the well-being of low- and middle-income, eastern and urban residents than high-income, midwest and rural residents.

## Figures and Tables

**Table 1:** Data Definition.

Variable Name		Label	Description	
Subjective Well-being (SWB)	Life satisfaction	satisfaction	Are you satisfied with your life?	1–5 denotes from very unsatisfied to very satisfied
	Positive emotions	posemotion	I feel joyful	1 = Never (less than one day) 2 = Sometimes (1–2 days) 3 = Often (3–4 days) 4 = Most of the time (5–7 days)
			I have a happy life	
	Negative emotions	negemotion	I am in a low spirit	1 = Most of the time (5–7 days) 2 = Often (3–4 days) 3 = Sometimes (1–2 days) 4 = Never (less than one day)
			I feel sad	
	Overall cognitive happiness valuation	coghapiness	How happy are you? (score)	0–10 denotes from very unhappy to very happy
Public health investment		phe	Per capita public health investment	
The disparity of public health investment		disparity	Individual relative deprivation index of public health investment	
Age		age	Age of respondents	
Gender		gender	1 = man; 0 = woman	
Ethnicity		ethnicity	1 = Han; 0 = another minority	
Marital status		marry	1 = in marriage; 0 = not in marriage	
Education		education	Years of schooling	
Registered permanent residence		identity	1 = rural; 0 = urban	
Relative income		income	1–5 denotes from very low-income level to a very high-income level in local	
Health status		health	1 = Excellent; 2 = Very good; 3 = Good; 4 = Fair; 5 = Poor	
Social status		status	1–5 denotes from very low to very high in local	
Politics status		party	1 = a member of Communist Party of China; 0 = not a member of Communist Party of China	
Household income level		lnphinc	The logarithm of per capita household income	
Family relationships		family	How many times do you usually have dinner with your family in one week	
Social network		lngift	The logarithm of gift expenditure	

**Table 2:** Summary statistics of sample data.

Variable Label	Mean	S.D.	Min	Median	Max
satisfaction	4.040	0.950	1	4	5
low_spirit	3.270	0.760	1	3	4
feel_sad	3.490	0.700	1	4	4
feel_joyful	2.900	0.930	1	3	4
happy_life	3.050	0.900	1	3	4
coghapiness	7.540	2.110	1	8	10
phe	1021	231.5	770.5	1002	2782
disparity	0.120	0.0500	0	0.110	0.210
age	47.89	15.32	16	48	96
gender	0.490	0.500	0	0	1
ethnicity	0.910	0.290	0	1	1
marry	0.870	0.340	0	1	1
education	7.630	4.960	0	9	22
identity	0.730	0.440	0	1	1
income	2.930	1.070	1	3	5
health	3.060	1.210	1	3	5
status	3.130	1.070	1	3	5
party	0.100	0.300	0	0	1
lnphinc	9.390	1.010	0.920	9.430	13.30
family	5.850	2.230	0	7	7
lngift	8	1.040	1.610	8.010	11.98

**Table 3:** Sample distribution of key variables.

Variable Label	Frequency	Percent	Cumulative Distribution
satisfaction			
1	416	1.81	1.81
2	675	2.93	4.74
3	5391	23.41	28.14
4	7644	33.19	61.33
5	8905	38.67	100
feel_joyful			
1	1681	7.3	7.3
2	6064	26.33	33.63
3	8082	35.09	68.72
4	7204	31.28	100
happy_life			
1	1336	5.8	5.8
2	4820	20.93	26.73
3	8230	35.73	62.46
4	8645	37.54	100
low_spirit			
1	834	3.62	3.62
2	1862	8.08	11.71
3	10,682	46.38	58.09
4	9653	41.91	100
feel_sad			
1	565	2.45	2.45
2	999	4.34	6.79
3	8072	35.05	41.84
4	13,395	58.16	100
coghapiness			
1	297	1.29	1.29
2	190	0.82	2.11
3	444	1.93	4.04
4	439	1.91	5.95
5	3609	15.67	21.62
6	1856	8.06	29.68
7	2461	10.69	40.36
8	5954	25.85	66.22
9	1850	8.03	74.25
10	5931	25.75	100

**Table 4:** Results of exploratory factor analysis (eigenvalue).

Factor	Principal Axis Factor Method (PF)	Iterative Principal Axis Factor Method (IPF)	Maximum Likelihood Factor Method (MLF)
Factor 1	1.74	1.97	1.86
Factor 2	0.32	0.54	0.54
Factor 3	0.26	0.49	—
Factor 4	−0.19	0.03	—
Factor 5	−0.21	0.01	—
Factor 6	−0.22	−0.0002	—

**Table 5:** The fitting degree of subjective well-being factor model.

Indicator	CFI	R^2^(CD)	RMSEA
Test result	0.999	0.596	0.016
judgement criteria	above 0.90		below 0.08

**Table 6:** Measured values of subjective well-being.

Region	Value Adjustment Factor	Standardized Value	Region	Value Adjustment Factor	Standardized Value
Beijing	11.97	89.58	Shandong	12.31	90.67
Tianjin	12.13	90.10	Henan	11.99	89.65
Hebei	12.02	89.74	Hubei	11.97	89.57
Shanxi	11.71	88.71	Hunan	11.76	88.89
Liaoning	12.14	90.11	Guangdong	11.40	87.71
Jilin	11.84	89.15	Guangxi	11.14	86.85
Heilongjiang	12.12	90.05	Chongqing	11.51	88.08
Shanghai	12.19	90.30	Sichuan	11.88	89.27
Jiangsu	12.02	89.73	Guizhou	10.91	86.11
Zhejiang	12.06	89.88	Yunnan	11.47	87.93
Anhui	11.94	89.47	Shanxi	11.22	87.12
Fujian	11.10	86.72	Gansu	11.32	87.44
Jiangxi	11.20	87.06	average	11.76	89.09

**Table 7:** MIMIC model estimation result.

Variable	(1)	(2)	(3)
	ML	ML + Robust	MLMV + Robust
A. Structural Equation			
phe	0.0007 ***	0.0007 ***	0.0006 ***
	(8.74)	(8.71)	(8.14)
disparity	8.1965 ***	8.1965 ***	7.2559 ***
	(7.43)	(7.41)	(6.89)
disparity2	−17.1280 ***	−17.1280 ***	−14.6824 ***
	(−5.35)	(−5.34)	(−4.79)
age	−0.0152 ***	−0.0152 ***	−0.0180 ***
	(−9.12)	(−8.54)	(−10.90)
age2	0.0002 ***	0.0002 ***	0.0002 ***
	(11.94)	(11.04)	(13.53)
gender	0.0439 ***	0.0439 ***	0.0325 ***
	(5.50)	(5.29)	(4.07)
ethnicity	−0.0332 **	−0.0332 **	−0.0116
	(−2.45)	(−2.39)	(−0.88)
marry	0.1456 ***	0.1456 ***	0.2002 ***
	(11.31)	(10.26)	(15.95)
education	0.0030 ***	0.0030 ***	0.0036 ***
	(2.81)	(2.65)	(3.37)
identity	−0.0634 ***	−0.0634 ***	−0.0742 ***
	(−6.22)	(−6.32)	(−7.75)
income	0.0817 ***	0.0817 ***	0.0828 ***
	(17.39)	(13.76)	(14.78)
health	−0.1406 ***	−0.1406 ***	−0.1401 ***
	(−36.74)	(−33.66)	(−35.85)
status	0.1030 ***	0.1030 ***	0.1110 ***
	(20.95)	(15.70)	(17.37)
party	0.0069	0.0069	0.0162
	(0.52)	(0.55)	(1.35)
lnphinc	0.0568 ***	0.0568 ***	0.0486 ***
	(12.33)	(11.80)	(11.29)
family	0.0204 ***	0.0204 ***	0.0208 ***
	(11.31)	(11.06)	(11.26)
lngift	0.0138 ***	0.0138 ***	0.0176 ***
	(3.51)	(3.49)	(4.50)
B. Measurement Equation			
satisfaction	1.0000	1.0000	1.0000
	(.)	(.)	(.)
low_spirit	0.6065 ***	0.6065 ***	0.5736 ***
	(35.27)	(25.45)	(26.48)
feel_sad	0.5855 ***	0.5855 ***	0.5713 ***
	(34.32)	(22.82)	(23.77)
feel_joyful	0.8538 ***	0.8538 ***	0.8175 ***
	(37.08)	(26.50)	(27.56)
happy_life	0.8814 ***	0.8814 ***	0.8597 ***
	(38.25)	(27.36)	(28.51)
coghapiness	2.4346 ***	2.4346 ***	2.4311 ***
	(53.09)	(40.95)	(44.13)
C. Fit Index			
N	23,031	23,031	27,062
RMSEA	0.041		
CFI	0.901		
SRMR	0.026	0.026	
R^2^(CD)	0.367	0.367	0.367

Note: (1) Z value in parentheses. (2) Asterisks indicate significance levels: ** represent significance levels of 10 percent; *** represent significance levels of 1 percent.

**Table 8:** MIMIC model estimation results of different income groups.

Variable	(1)	(2)	(3)	(4)	(5)
	MLMV + Robust				
	Low	Low–Middle	Middle	Upper Middle	High
A. Structural Equation					
phe	0.0006 ***	0.0007 ***	0.0005 ***	0.0005 ***	0.0003 ***
	(4.43)	(3.07)	(2.81)	(3.42)	(2.67)
disparity	7.6012 ***	9.0059 ***	9.1372 ***	5.6687 ***	2.7784
	(4.73)	(3. 87)	(4.43)	(2.64)	(1.46)
disparity2	−14.8418 ***	−19.4051 ***	−23.0571 ***	−10.6304 *	−3.3760
	(−3.04)	(−2.78)	(−3.86)	(−1.69)	(−0.59)
age	−0.0161 ***	−0.0203 ***	−0.0129 ***	−0.0162 ***	−0.0093 **
	(−5.17)	(−4.97)	(−3.55)	(−4.35)	(−2.50)
age2	0.0002 ***	0.0003 ***	0.0002 ***	0.0002 ***	0.0001 ***
	(6.29)	(6.16)	(5.25)	(5.68)	(3.85)
gender	0.0358 **	0.0707 ***	0.0383 **	0.0612 ***	0.0287
	(2.25)	(3.64)	(2.24)	(3.43)	(1.63)
ethnicity	−0.0822 ***	−0.0296	−0.0236	−0.0030	0.0542
	(−3.56)	(−0.96)	(−0.75)	(−0.08)	(1.43)
marry	0.1440 ***	0.1437 ***	0.1523 ***	0.1308 ***	0.1794 ***
	(5.96)	(4.50)	(5.47)	(4.39)	(6.13)
education	0.0061 ***	0.0066 ***	0.0026	−0.0006	−0.0026
	(3.00)	(2.58)	(1.12)	(−0.24)	(−1.02)
identity	−0.1321 ***	−0.0102	−0.0190	−0.0822 ***	−0.0570 ***
	(−5.19)	(−0.33)	(−0.93)	(−4.13)	(−2.73)
income	0.1035 ***	0.0470 ***	0.0802 ***	0.0737 ***	0.0894 ***
	(12.78)	(4.71)	(8.50)	(7.09)	(7.99)
health	−0.1318 ***	−0.1435 ***	−0.1513 ***	−0.1473 ***	−0.1259 ***
	(−20.40)	(−17.50)	(−19.99)	(−17.69)	(−14.63)
status	0.1038 ***	0.1035 ***	0.0910 ***	0.0992 ***	0.1158 ***
	(12.64)	(9.79)	(9.51)	(9.66)	(10.12)
party	0.0242	−0.0107	0.0302	−0.0146	0.0230
	(0.77)	(−0.27)	(1.01)	(−0.52)	(0.98)
lnphinc	0.0347 ***	0.0427	0.0564	0.0679 *	0.0618 ***
	(4.06)	(0.91)	(1.47)	(1.86)	(3.31)
lngift	0.0218 ***	0.0001	−0.0057	0.0171 *	0.0254 ***
	(2.83)	(0.1)	(−0.67)	(1.92)	(2.78)
family	0.0147 ***	0.0149 ***	0.0218 ***	0.0248 ***	0.0268 ***
	(3.93)	(3.30)	(5.95)	(6.30)	(6.67)
N	6574	3967	4572	4236	3682

Note: (1) Z value in parentheses. (2) Asterisks indicate significance levels: * represent significance levels of 10 percent; ** represent significance levels of 10 percent; *** represent significance levels of 1 percent.

**Table 9:** MIMIC model estimation results of different areas.

Variable	(1)	(2)	(3)	(4)
	MLMV + Robust			
	East	Midwest	Rural	Urban
phe	0.0005 ***	0.0004 ***	0.0006 ***	0.0005 ***
	(4.88)	(2.98)	(6.83)	(5.55)
disparity	6.4905 ***	9.8656 ***	8.2085 ***	5.1556 ***
	(3.89)	(7.19)	(7.2)	(3.9)
disparity2	−13.9285 ***	−26.3968 ***	−18.0201 ***	−8.3904 ***
	(−2.83)	(−6.00)	(−5.38)	(−2.13)
age	−0.0146 ***	−0.0150 ***	−0.0152 ***	−0.0119 ***
	(−5.78)	(−7.00)	(−7.74)	(−3.98)
age2	0.0002 ***	0.0002 ***	0.0002 ***	0.0002 ***
	(7.54)	(9.21)	(9.99)	(5.76)
gender	0.0649 ***	0.0322 ***	0.0417 ***	0.0513 ***
	(5.33)	(3.10)	(4.41)	(3.57)
ethnicity	−0.0080	−0.0607 ***	−0.0461 ***	0.0088
	(−0.3)	(−3.72)	(−3.07)	(0.27)
marry	0.1856 ***	0.1219 ***	0.1454 ***	0.1375 ***
	(9.15)	(7.46)	(9.54)	(5.94)
education	0.0019	0.0031 ***	0.0045 ***	−0.0009
	(1.13)	(2.30)	(3.6)	(−0.44)
identity	−0.0682 ***	−0.0645 ***	-	-
	(−4.5)	(−4.64)	-	-
income	0.0855 ***	0.0790 ***	0.0848 ***	0.0673 ***
	(12.12)	(13.77)	(16.06)	(7.78)
health	−0.1406 ***	−0.1396 ***	−0.1403 ***	−0.1401 ***
	(−25.09)	(−29.81)	(−32.95)	(−19.87)
status	0.1017 ***	0.1036 ***	0.0977 ***	0.1182 ***
	(14.15)	(17.29)	(17.85)	(13.35)
party	0.00007	0.0161	0.0175	−0.0002
	(0.00)	(0.93)	(0.94)	(−0.01)
lnphinc	0.0601 ***	0.0465 ***	0.0572 ***	0.0564 ***
	(8.40)	(7.62)	(10.85)	(6.06)
family	0.0208 ***	0.0196 ***	0.0181 ***	0.0270 ***
	(7.56)	(8.37)	(8.61)	(8.03)
lngift	0.0084	0.0200 ***	0.0058	0.0365 ***
	(1.4)	(3.85)	(1.26)	(4.86)
N	9162	13,869	16,917	6114

Note: (1) Z value in parentheses. (2) Asterisks indicate significance levels: *** represent significance levels of 1 percent.

## Data Availability

All data analyzed in this study are publicly available from Peking University Open Research Data Platform (https://opendata.pku.edu.cn/ accessed on 28 January 2022).
